# Iterative Development and Applicability of a Tablet-Based e-Coach for Older Adults in Rehabilitation Units to Improve Nutrition and Physical Activity: Usability Study

**DOI:** 10.2196/31823

**Published:** 2022-03-16

**Authors:** Lisa Happe, Marie Sgraja, Andreas Hein, Rebecca Diekmann

**Affiliations:** 1 Department of Health Services Research, Assistance Systems and Medical Device Technology Carl von Ossietzky University Oldenburg Oldenburg Germany

**Keywords:** older adults, rehabilitation, physical activity, nutrition, e-coach, usability testing, tablet computers, health behavior, mobile phone

## Abstract

**Background:**

Maintaining nutrition and exercise strategies after rehabilitation can be difficult for older people with malnutrition or limited mobility. A technical assistance system such as an e-coach could help to positively influence changes in dietary and exercise behavior and contribute to a sustainable improvement in one’s nutrition and mobility status. Most apps do not provide a combination of nutrition and exercise content. In most cases, these apps were evaluated with healthy individuals aged <70 years, making transferability to vulnerable patients, with functional limitations and an assumed lower affinity for technology, in geriatric rehabilitation unlikely.

**Objective:**

This study aims to identify the potential for optimization and enhance usability through iterative test phases to develop a nutrition and mobility e-coach suitable for older adults (≥65 years) based on individual health behavior change stages in a rehabilitation setting.

**Methods:**

Iterative testing was performed with patients aged ≥65 years in a rehabilitation center. During testing, participants used an e-coach prototype with educational elements and active input options on nutrition and mobility as a 1-time application test. The participants performed navigation and comprehension tasks and subsequently provided feedback on the design aspects. Hints were provided by the study team when required, documented, and used for improvements. After testing, the participants were asked to rate the usability of the prototype using the System Usability Scale (SUS).

**Results:**

In all, 3 iterative test phases (T1-T3) were conducted with 49 participants (24/49, 49% female; mean 77.8, SD 6.2 years). Improvements were made after each test phase, such as adding explanatory notes on overview screens or using consistent chart types. The use of the user-centered design in this specific target group facilitated an increase in the average SUS score from 69.3 (SD 16.3; median 65) at T1 to 78.1 (SD 11.8; median 82.5) at T3. Fewer hints were required for navigation tasks (T1: 14.1%; T2: 26.5%; T3: 17.2%) than for comprehension questions (T1: 30.5%; T2: 21.6%; T3: 20%). However, the proportion of unsolved tasks, calculated across all participants in all tasks, was higher for navigation tasks (T1: 0%, T2: 15.2%, T3: 4.3%) than for comprehension tasks (T1: 1.9%, T2: 0%, T3: 2.5%).

**Conclusions:**

The extensive addition of explanatory sentences and terms, instead of shorter keywords, to make it easier for users to navigate and comprehend the content was a major adjustment. Thus, good usability (SUS: 80th-84th percentile) was achieved using iterative optimizations within the user-centered design. Long-term usability and any possible effects on nutritional and physical activity behavior need to be evaluated in an additional study in which patients should be able to use the e-coach with increasing independence, thereby helping them to gain access to content that could support their long-term behavior change.

## Introduction

### Background

Different demographic, clinical, biological, and lifestyle factors contribute to the development of frailty and sarcopenia in older populations. The accumulation of these risk factors leads to a reduction in resistance to health stressors. In addition to a decline in independence, there is an increased risk of falls and mortality [1,2]. In many older people, malnutrition and reduced physical activity are associated with each other [3]. The performance of physical exercises in combination with a protein-rich diet is a promising approach for the prevention and treatment of frailty [1]. Such treatments can be provided as part of outpatient or inpatient geriatric rehabilitation, although inpatient geriatric rehabilitation is more common in Germany. In the context of geriatric rehabilitation, patients are treated by a multidisciplinary team, which focuses on the individual needs and abilities of the patient [4,5]. However, long-term maintenance by older adults after rehabilitation is often unsuccessful. Negative influences on adherence, such as sudden changes in health status, a lack of interest or motivation, low self-efficacy, or low expectations of improvement [6,7] are likely to be factors affecting this lack of success.

Technical assistance systems, such as health apps, could help ensure that dietary and exercise behaviors are implemented and changed after rehabilitation. In a survey in Germany with older adults (aged >65 years) in 2020, up to 22% reported that they used health apps to track fitness data, and 16% used apps to obtain information about health, fitness, and nutrition topics. However, the proportion of seniors interviewed who could envision using such apps was more than twice as high for both types of health apps [8]. Factors that influence the acceptance of health apps should be considered in an effort to encourage older adults to use such apps. Barriers to the acceptance of health apps include a lack of trust in health apps, privacy concerns, and fear of misdiagnosis. In addition, older people who generally use apps, but who have no experience using health apps, reported a lack of health app usability and low self-confidence as reasons for poor acceptance [9]. In addition to improving older people’s access to technology, it is also essential that health apps are valid, reliable, and based on current scientific evidence. It is recommended to increase the involvement of older people in the design, conceptualization, and testing of such apps [10]. Most scientifically developed health apps were evaluated with individuals aged up to 70 years or with older adults without health impairments [11-13].

Apps are one type of technical tool for improving, assisting, or supporting people. The various realizations, such as tele-visits, exergames, or health websites, can be summarized under the term eHealth. A review of the use of eHealth in the context of geriatric rehabilitation revealed that most studies (68%) involved people with neurological diseases. In addition, only 8% of all identified studies assessed the use of health apps as an eHealth intervention. However, the results on the applicability of eHealth with the target group indicated that interventions are feasible if adequate training takes place, and if the eHealth intervention is simple and has good usability [14]. For example, a study on telerehabilitation via a website compared with conventional rehabilitation after a hip fracture showed that patients in the telerehabilitation group achieved better functional scores than those in the control group [15]. In addition, in a study by Bean et al [16], the implementation of a 12-month web-based training program using a tablet and supervised by a physiotherapist led to a significant reduction in emergency department visits and hospital admissions in older adults with mobility impairments. However, there is also evidence that the more severe the health limitations of older people, the less willing they are to use eHealth [17]. A systematic review of the use of health apps to improve dietary behavior and nutrition-associated outcomes was unable to find any studies that focused on the use of such apps by people aged >70 years [18].

As previously described, a nutrition intervention in combination with an exercise intervention could lead to more significant effects than would an exercise intervention alone in older people with frailty or sarcopenia [2]. An app that offers coaching in the sense of providing information and feedback on physical activity and nutrition topics, thus supporting behavior change or the maintenance of newly adopted behaviors and tailored to the needs of older people, could therefore be a promising approach to making a rehabilitation program even more effective and sustainable. The use of the app should be continued after rehabilitation and thus provide support in everyday life in the uptake, implementation, and maintenance of the recommendations in the area of nutrition and physical activity.

To develop an age-adapted device and health app (e-coach) for older adults with deficits in nutritional status and physical activity needs, we followed the German International Organization for Standardization 9241-210:2019 *Ergonomics of human–system interaction—Part 210: Human-centered design of interactive systems* [19] and the user-centered design process [20].

These concepts first require an analysis of the context of use and, as a next step, a specification of the use requirements. The context of use was described in a previous study by performing and analyzing focus groups with older adults as well as experts [21]. In all, 3 focus groups with patients and relatives (10/17, 65% female; 16/17, 94% in a 70- to 99-year–age category) and 1 focus group with experts (2 dieticians and 1 physiotherapist) were held in a geriatric rehabilitation center. Interviews held with the focus groups were recorded, transcribed verbatim, and analyzed using content analysis. Both patients and therapists mentioned very similar points as relevant topics for e-coaches. Examples of the aspects mentioned included information about nutrition in advanced age, macronutrients, fluid intake, nutrition myths, physical activity recommendations for older adults, guidance in performing physical exercises, information on goal setting, the risk of falling, and adherence to physical activities. However, individual perceptions of the need for further information varied widely.

The information gained was used to derive the user and design requirements for the e-coach. For geriatric patients in rehabilitation, educative content from the areas of nutrition and physical activity focused on the changes and demands of aging that should be included in the e-coach. The older adults would also like the e-coach to be able to provide them with exercises and thus support them in their training. The feedback and evaluation of input regarding nutrition and exercise are described as helpful but should not be an admonition. The results indicate that, as many patients in this age group have little experience with technology and usually use other sources of information, it is important to develop a nutrition and mobility e-coach, particularly given the easy handling and provision of clear information to individual users on the advantages of the e-coach. It is also important for older adults to avoid barriers, such as small font, low video volume, or poor contrast.

The e-coach needs to be integrated into users’ daily lives without stressing or restricting them. Moreover, it must be possible to adapt the content to the physical abilities of the users, and because of the heterogeneity of older people in terms of previous knowledge and willingness to change their behavior, appropriate strategies should be used. A recent umbrella review of eHealth interventions suggested that applications involving behavior change techniques may have promising effects on physical activity, sedentary behavior, and healthy eating. However, it is not yet known which theoretical construct is the most effective [22]. In this study, the transtheoretical model of behavior change (TTM) was used as the underlying psychological construct for the e-coach. The patient was categorized into one of the five TTM phases reflecting their readiness for change: (1) precontemplation, (2) contemplation, (3) planning, (4) action, and (5) maintenance. In the first phase (precontemplation), there is no awareness of the problem or intention to act yet, whereas in the fourth stage (action), the desired behavior is already specifically being executed. Different strategies are used at each phase to achieve or sustain targeted behavior [23]. The model was first developed in the context of substance abuse treatment; however, it has also been applied to other health behavior change processes, such as increasing physical activity [24,25]. A recent systematic review on the use of the TTM in programs designed to improve physical activity in older people showed positive effects on relevant parameters, such as the reduction of sedentary behavior, the increase in activity time per week, an increase in the number of steps, and an increase in the daily total of moderate to vigorous activity time [26]. In another study that used an app to increase physical activity in healthy older adults, the TTM was also used and positive effects on exercise adherence and walking speed were observed [13].

On the basis of the findings from the focus groups, it was possible to further differentiate the settings for teaching the use of the e-coach. To give older adults time to familiarize themselves with the system and generally introduce them to its use, this introduction should already take place in the rehabilitation center. In a real health care situation, it would be easier to explain the technology to the patient; in case of questions or if further explanations are necessary, it would be more uncomplicated to address these points in a personal appointment. In addition, patients would also be able to repeat relevant content that they may not have been able to remember completely from the seminars at their own pace. Therapists should have the possibility to adapt the e-coach to the needs of the patients and their TTM phase. As, in the context of rehabilitation, the therapies take place directly between the physiotherapist or nutritionist and the patient, and the interventions are also strongly influenced by the interactions between the professionals and patients, complete automation of the e-coach would not be efficient. Adaptations of the e-coach to the patient’s previous knowledge and support needs should therefore be made by a physiotherapist or a nutritionist.

### Objectives

This paper aims to describe the design process and an iterative evaluation of the developed content. The aim of this study is to identify optimization potentials and enhance usability through iterative test phases to develop a nutrition and mobility e-coach based on individual health behavior change stages, usable for older adults (≥65 years) in a rehabilitation setting.

## Methods

### Study Design

The e-coach prototypes were evaluated with older adults in 3 iterative test phases, using a between-subject design. User experience was reflected by the System Usability Scale (SUS) [27] and participants’ comments, which were made while thinking aloud during the tests.

To detect and analyze usability problems in more detail, at least 10 patients were included in each iterative test phase [28]. Then, based on the feedback, improvements were made and the prototypes were evaluated again with the target group. The opinions of other relevant stakeholders (physiotherapists and nutritionists) were taken into account throughout the design process by involving professionals from these disciplines in the study team.

### Ethics Approval

The study was approved by the Ethical Review Board of the Carl von Ossietzky University Oldenburg (registration number: 2018–132). We conducted the study in accordance with the Declaration of Helsinki and the underlying data protection regulation.

### Participants

Patients in rehabilitation, from geriatric and cardiology wards, were eligible based on the following inclusion criteria: (1) participants aged ≥65 years and (2) participants were able to speak and understand German. Exclusion criteria were (1) severe visual impairment (eg, inability to read large font on a screen), (2) severe hearing impairment (eg, deafness), or (3) inability to understand study information and provide informed written consent (eg, aphasia or severe cognitive impairment or dementia). Participants were recruited by placing flyers in the patients’ wards.

### Tablet App (e-Coach)

The e-coach screens were designed in Adobe XD (version 34; Adobe Systems) for a 10-inch tablet in landscape mode. Design guidelines for apps for older adults were used to take into account specific requirements, such as a decline in vision or decreased motor abilities when developing the prototype [29,30]. To ensure a linear navigation structure, the position and design of the navigation buttons were always identical. The only gesture needed was to tap a button to minimize the number of necessary gestures. The structure of different screens containing the same type of information provision (eg, videos or texts) was kept identical.

**Figure 1:**
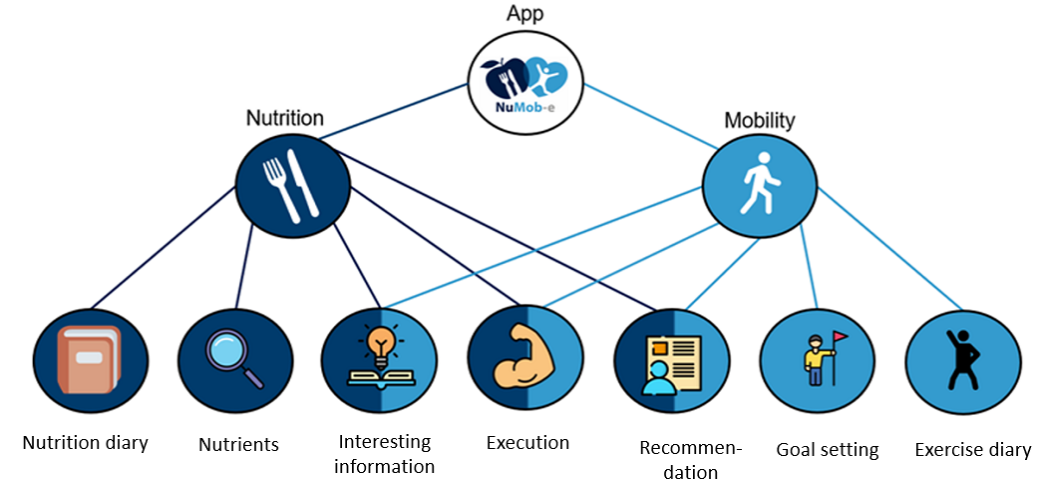
Structure of the e-coach modules.

The e-coach contained two main topics: *mobility* and *nutrition*. Both topics offered five modules in total, which were identified from previous focus group discussions with patients and experts. The e-coach was designed to support behavior change in older, vulnerable patients in rehabilitation by providing information, education about risk factors, and strategies for implementing and maintaining nutrition and physical activity recommendations. Different elements, such as videos, texts, or quizzes, were used to provide the information. In addition, the e-coach included educational elements and active input options, such as feedback on nutrient intake through entries in a nutrition diary or instructions and documentation of physical exercises, and an overview of the achievement of exercise goals. The modules entitled *interesting facts*, *recommendations*, and *execution* were elements of both main themes, although with different subthemes (eg, content about fall risk factors or strategies to promote the intake of fluids). The modules and their content differed according to the phase of the TTM. For example, by focusing more on educational content in the precontemplation and contemplation phases, strategies to increase problem awareness, environmental re-evaluation, and emotional experience are promoted, whereas, in the planning, action, and maintenance phases, more content was introduced that enabled the maintenance of new behaviors, such as enhancing self-efficacy or offering specific action guidance. The module content and the composition of modules for each TTM phase were compiled in advance by members of the study team who have expertise in physiotherapy and nutritional therapy. The content of the nutrition modules was based on the recommendations of the European Society for Clinical Nutrition and Metabolism guidelines on clinical nutrition and hydration in geriatrics [31], as well as on the information provided in brochures issued by the IN FORM initiative of the *Deutsche Gesellschaft für Ernährung* (German Nutrition Society) entitled *Essen und Trinken im Alter* (Eating and drinking in old age) [32] and *Mangelernährung im Alter* (Malnutrition in old age) [33]. The content of the physical activity modules was mainly based on the national recommendations for physical activity and physical activity promotion, the *Älter werden in Balance* (Getting Older in Balance) program of the Federal Center for Health Education and the recommendations from the IN FORM initiative run by *Bundesarbeitsgemeinschaft der Seniorenorganisationen* (German National Association of Senior Citizens’ Organizations) [34]. Physical exercises were based on exercises from the Otago Exercise Program [35], Lifestyle-integrated Functional Exercise-Program [36], and home-based older people’s exercise [37] programs, which were developed especially for older people and people at risk of falling. Automated adaptation of module content was not part of the e-coach. The results of the focus groups did not indicate that this would be a requirement for therapists for such apps. Moreover, the app should be able to be used as support and an addition within the scope of therapies in rehabilitation and in further outpatient care. Therefore, the use should be embedded in the context of the therapy situation, and the assessment of the TTM phase should not be done completely automatically but by the therapist in interaction with the patient. Therefore, the TTM phase is determined as described below and then set in the app. In the context of this study, no direct association between the TTM phase and the ability to perform tasks in the app was assumed, as it was a 1-time application test with specific task instructions to the participants. The general structure of the e-coach is shown in Figure 1.

### Procedure

#### Overview

Data were collected in the form of in-person testing in patients’ rooms at the rehabilitation center. After the patients had been informed of the content and the procedure of the study by a study team member (LH or MS) and had signed the informed consent, a survey was conducted on sociodemographic data, data on nutrition and physical activity, the phases of behavioral change, and technology commitment. A usability test was subsequently performed. The entire process of data collection took approximately 45 minutes to 60 minutes per patient.

#### Evaluation of Nutritional Data and Physical Activity

Nutritional status was assessed using the Mini Nutritional Assessment-Short Form (MNA-SF) [38] and a survey of nutritional behavior based on an eating protocol for a typical day. The data from the eating protocol were compared with the recommendations of the German Nutrition Society for people aged >65 years [39]. Physical activity was evaluated using the Physical Activity Scale for the Elderly (PASE) [40]. The intensity, type, and duration of the activities described were compared with the German national recommendations for physical activity in older adults [41].

#### Phases of Behavior Change (TTM)

Patients were classified separately into the TTM for physical activity and nutrition based on the data from the dietary protocol and the PASE. Patients who did not achieve the defined target criteria in the areas of physical activity or nutrition were asked whether they had thought about changing their behavior (contemplation) and, if so, whether they had already planned to do so in any specific way (planning). Depending on their answers, patients were then categorized into the phases of precontemplation, contemplation, or planning. Patients who were already performing the target behavior were asked whether they had been doing so for a short time (action) or for a longer period (maintenance). On the basis of their answers, the patients were classified into the phases of action or maintenance.

#### Technology Commitment

In addition, the patients’ technology commitment was assessed using a questionnaire developed by Neyer et al [42]. In this questionnaire, technology commitment was measured using a 5-point Likert scale with 12 items. The items covered statements about personal contact, interest, and the use of technologies in general [42].

#### Usability Task

The test procedure was explained in detail, and the contents and navigation options were shown in advance. Before testing the usability task, the patients were told that the aim of the study was not to test their abilities, but the quality and usability of the e-coach [43]. Moreover, the patients were able to choose whether they wanted to be interviewed about content from one main topic only (nutrition or mobility) or about both topics. The status of development and the general structure of the mock-ups from the 2 areas were identical, but the content differed on account of topics. All elements that could be used by patients in the final e-coach were tested; however, not every screen was tested, instead, a transferability of findings was assumed.

Testing also took place in the patient’s room at the rehabilitation center. During the test, the examiner (LH or MS) and the participant sat at a table. The tablet with the app could be placed on the integrated stand of the device, placed on the table, held in the hand, or laid down by the participant. The examiner read the tasks to the participant and then observed the participant.

Usability tasks in three different domains were defined and used for each iterative testing period: navigation, comprehension, and design. Navigation tasks were used to determine whether users were able to find their way through the e-coach and use the buttons correctly. In the case of quizzes, we tested whether the screens were structured such that they could be used successfully by the participants. On quiz screens, the question was highlighted at the top of the screen (eg, What is the recommended minimum number of small portions of dairy products to eat per day?) and below it, two to three answer options were shown (eg, You should eat at least two servings of dairy products per day and You should eat at least four servings of dairy products a day) along with a prompt (press the correct answer). Comprehension tasks were intended to test whether screen content and information were correctly interpreted and understood. The aim of the design questions was to identify visual barriers such as a font that was too small or an acoustic problem, such as an extremely fast rate of speech in videos. An example of questions and tasks is shown in Table 1 and Multimedia Appendices 1 and 2.

**Table 1:** Task types in the different iterative testing phases as the total number of tasks and percentages per iterative testing phase.

Task type			Navigation task, T1^a^, n (%)	Navigation task, T2^b^, n (%)	Navigation task, T3^c^, n (%)	Comprehension question, T1, n (%)		Comprehension question, T2, n (%)		Comprehension question, T3, n (%)	
**Navigation**							N/A^d^		N/A		N/A
	Next screen (1 screen)		16 (57)	5 (24)	3 (30)						
	Further screen (≥2 screens)		4 (14)	5 (24)	4 (40)						
	Use of back button		5 (18)	5 (24)	2 (20)						
	Use of different tabs (text elements)		2 (7)	2 (10)	0 (0)						
	Use of the help button		0 (0)	3 (14)	1 (10)						
	Use of quizzes		1 (4)	0 (0)	0 (0)						
	Use of the exercise diary		0 (0)	1 (5)	0 (0)						
**Comprehension**			N/A	N/A	N/A						
		Purpose of the screen				5 (28)		2 (20)		1 (25)	
		Foresight of content				3 (17)		0 (0)		1 (25)	
		Nutrition diary				1 (6)		0 (0)		0 (0)	
		Interpretation of content				7 (39)		8 (80)		2 (50)	
		Understanding of quizzes				2 (11)		0 (0)		0 (0)	

^a^T1: iterative phase 1.

^b^T2: iterative phase 2.

^c^T3: iterative phase 3.

^d^N/A: not applicable.

#### Evaluation of Usability

The results from the usability tasks were reported in three different categories (success rate, number of hints, and content of hints) to evaluate usability problems in more detail. The performance of the particular task was evaluated in the categories of *successful* and *unsuccessful*. Furthermore, the content of the hints given and the number of hints given were recorded.

For the usability test, patients were instructed to simultaneously speak their thoughts aloud while performing the tasks. The concurrent think-aloud method was intended to immediately identify and specify problems for older adults using the e-coach [44]. Attention was paid not to interrupt patients while they were still thinking or looking for an answer. However, if the participant said that they were stuck or if they were obviously having difficulties, (eg, the participant looked around for help for a longer period or became increasingly nervous) a hint was given. These hints mostly consisted of a slight rephrasing of the question or a request to the participant to read through the contents of the screen again. If a hint had to be given, this was noted for the relevant task, and the number of times hints were given was counted.

The tasks in the test sequence were always carried out in the same order, but patients had the option of skipping tasks at any time or stopping the usability test. The tests and verbal feedback during the tasks were recorded by taking notes.

After the usability test, patients were finally interviewed using the SUS. The questionnaire contained 10 statements about the usability of a system rated on a 5-point Likert scale. The values of individual items were added together and then multiplied by 2.5, resulting in a score between 0 and 100. The results provided a general overview of product usability [27].

#### Iterative Process

**Figure 2:**
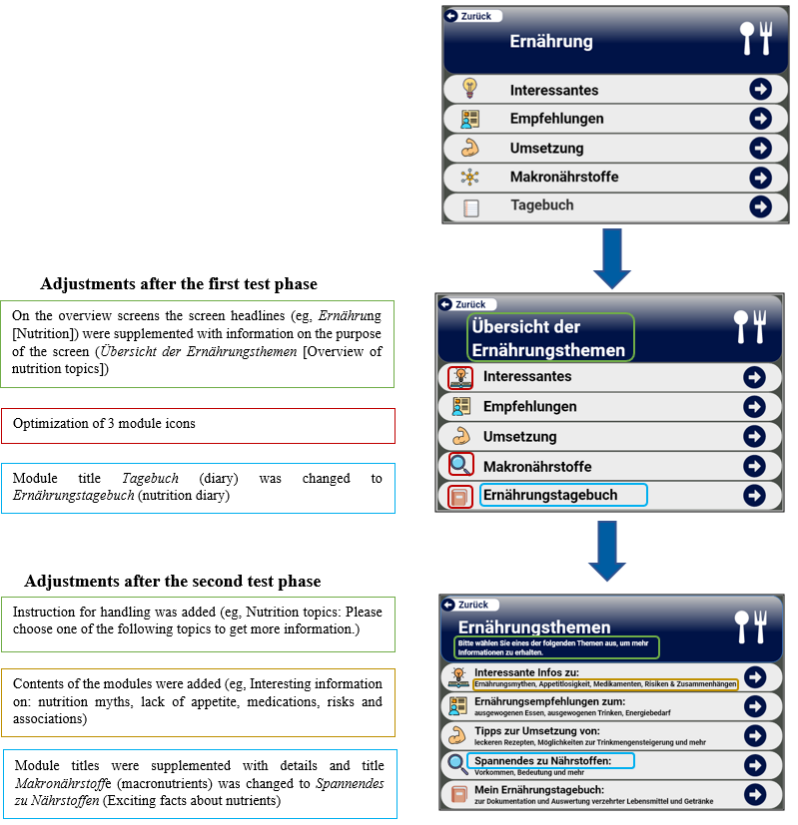
German original version of screens from the 3 iterative test phases. Explanations and translations of changed elements based on the results of the test phase are shown in the text boxes.

A total of 3 iterative test phases were used. Before each test phase, we refined the content, design, and potential functionality of the elements. The first test phase mainly tested the basic functionality of the chosen navigation structure with the target group, with *simpler* tasks such as finding the next page; in phases 2 and 3, we increased the number of navigation steps required in some tasks and tested other functions such as the use of the help button. To minimize the contact time and number of contacts with the study team in the context of the increasing incidence of COVID-19 in our region in Germany, it was decided to test the optimized elements and new content in the nutrition section only after completion of the second iterative test phase. As no further problems were found concerning the exercise part, but some were found concerning navigation from screens or the interpretation of nutritional diagrams, the last iterative phase focused on the questions from the nutrition part. Figure 2 shows an example of screenshots from version 1 to the final version. Multimedia Appendices 1 and 2 show click routes from the areas of nutrition and exercise, respectively.

### Data Analysis

Statistical analyses were performed using the software SPSS (version 27.0; SPSS Inc).

Participants’ characteristics, success rates of the tasks, and SUS scores were analyzed using descriptive statistics; they were presented as frequencies, means, SDs, and percentages.

The hints given during the usability test were presented as the total number of hints required for this task type in the iterative phase. The total number of unsolved tasks and percentages of unsolved tasks for all participants that performed this task type in the iterative phase were also reported.

The notes from the concurrent thinking aloud during the usability test were used to derive aspects that the participants noticed during the test. These aspects were discussed by the study team (LH and MS) to identify specific problems in the tasks and to derive possibilities for optimization.

An explorative ANOVA was conducted to compare the SUS scores among the 3 iterative test phases. The robust Welch *F* test was used if the assumption of homogeneity of variances was violated or if the data were not normally distributed.

The group was divided into 2 equally sized subgroups to test for group differences in successful task completion. Half of the participants who performed the tasks better were compared with the other half. The variables of age, sex, BMI, MNA-SF classification, technology affinity, TTM phase nutrition, TTM phase mobility, and SUS score were tested for group differences. The explorative analysis was performed using the Mann–Whitney *U* test for ordinal and not normally distributed variables, and the chi-square test was performed for nominal variables. Statistical significance was set at *P*<.05, for all explorative analyses.

## Results

### Characteristics of Participants

A total of 49 patients who were aged 66-94 years (24/49, 49% female; mean 77.8, SD 6.2 years) participated in the study. Patient characteristics per iterative test phase are presented in Table 2. An example of screens and changed elements based on the results of the iterative test phases are shown in Figure 2.

**Table 2:** Overview of participants’ characteristics within each iterative test phase (N=49).

Characteristics			Iterative phase 1				Iterative phase 2				Iterative phase 3^a^
			Nutrition		Mobility		Nutrition		Mobility		Nutrition
**Participants^b^**											
	Total, n	15		12		13		13		12	
	Female, n (%)	8 (53)		6 (50)		9 (69)		5 (39)		5 (42)	
BMI (kg/m^2^), mean (SD)			26.3 (4.4)		26.6 (5.6)		27.6 (5.8)		26.6 (4.6)		27.6 (4.3)
Age (years), mean (SD)			79.1 (6.8)		78.4 (5.5)		78.5 (7.3)		76.3 (6.8)		76.8 (5.2)
**TTM^c^phase, n (%)**											
	Precontemplation	3 (20)		1 (8)		4 (31)		0 (0)		3 (25)	
	Contemplation	2 (13)		5 (42)		3 (23)		5 (39)		2 (17)	
	Planning	4 (27)		3 (25)		1 (8)		4 (31)		5 (42)	
	Action	0 (0)		1 (8)		2 (15)		1 (8)		0 (0)	
	Maintenance	6 (40)		2 (17)		3 (23)		3 (23)		2 (17)	
**TC^d^, mean (SD)**											
	Total score (12-60 points)	36.6 (11.3)		39.3 (13.4)		38.7 (11.4)		43.7 (6.9)		39.4 (11.9)	
	Points^e^	3.1 (1.0)		3.4 (1.6)		3.3 (1.1)		3.7 (0.6)		3.3 (1.0)	

^a^Only tests in the nutrition section were performed to keep contact times and numbers low in the context of increasing COVID-19 incidence in the region.

^b^Different sample sizes within nutrition and mobility owing to the patient’s choice option to participate only in one main theme or in both main themes.

^c^TTM: transtheoretical model of behavior change.

^d^TC: technology commitment (Neyer et al [42]).

^e^Average Likert scale points per item.

Navigation tasks required fewer hints (14.1%-26.5%) per task than comprehension tasks (20%-30.5%) in all iterative test phases (Table 3). The percentage of tasks that participants were unable to successfully complete was 0% for the navigation tasks in iterative test phase 1; 15.2% in iterative test phase 2; and 4.3% in iterative test phase 3. For the comprehension tasks, participants were unable to complete 1.9% of the total tasks in iterative test phase 1; 0% of the tasks in iterative test phase 2; and 2.5% of the tasks in iterative test phase 3 despite receiving hints. An overview of the tasks that could partially not be solved and the respective optimizations is provided in Multimedia Appendix 3.

**Table 3:** Participants’ performance in the different navigation and comprehension tasks in the iterative test phases.

Tasks		Iterative phase 1				Iterative phase 2				Iterative phase 3		
		Total tasks^a^, N	Hints^b^, n (%)	Fail^c^, n (%)	Total tasks, N		Hints, n (%)	Fail, n (%)	Total tasks, N		Hints, n (%)	Fail, n (%)
**Navigation (total tasks)**		177	25 (14)	0 (0)	204		54 (27)	31 (15)	93		16 (17)	4 (4)
	Next screen (1 screen)	105	15 (14)	0 (0)	49		10 (20)	8 (16)	29		4 (14)	1 (4)
	Further screen (≥2 screens)	21	3 (14)	0 (0)	54		20 (37)	10 (19)	37		8 (22)	3 (8)
	Use of back button	32	6 (19)	0 (0)	55		10 (18)	6 (11)	18		1 (6)	0 (0)
	Use of tab layout (text elements)	12	1 (8)	0 (0)	9		2 (22)	2 (22)	0		0 (0)	0 (0)
	Use of the help button	0	0 (0)	0 (0)	28		10 (36)	5 (18)	9		3 (33)	1 (11)
	Use of quizzes	7	0 (0)	0 (0)	0		0 (0)	0 (0)	0		0 (0)	0 (0)
	Use of exercise diary	0	0 (0)	0 (0)	9		2 (22)	1 (11)	0		0 (0)	0 (0)
**Comprehension (total tasks)**		105	32 (31)	2 (2)	74		16 (22)	0 (0)	40		8 (20)	1 (3)
	Purpose of screen	33	11 (33)	0 (0)	26		5 (19)	0 (0)	9		1 (11)	0 (0)
	Foresight of content	15	6 (40)	0 (0)	0		0 (0)	0 (0)	12		2 (17)	0 (0)
	Nutrition diary	8	6 (75)	2 (25)	0		0 (0)	0 (0)	0		0 (0)	0 (0)
	Interpretation of content	38	7 (18)	0 (0)	72		11 (15)	0 (0)	19		5 (26)	1 (5)
	Comprehension of quizzes	11	2 (19)	0 (0)	0		0 (0)	0 (0)	0		0 (0)	0 (0)

^a^Total number of tasks performed by all participants per iterative phase.

^b^Summed up the number and percentage of required hints for all participants for this task type in the iterative phase.

^c^Total number and percentage of unsolved tasks in all participants who performed this task type in the iterative phase.

Most participants understood how to use the buttons correctly. In 86.9% of all tests, participants consistently selected the arrow button for navigation to the next screen as intended. When navigating back to previous screens, this was done completely correctly in 84.7% of all tasks.

Many participants were able to successfully interpret the active input options such as the drinking protocol (11/15, 73%), the diagram of consumed food groups (9/10, 90%), and the exercise diary (7/8, 88%).

Some participants had difficulties in interpreting the content they would expect to find based on the names of the modules or the themes. The contents that needed to be optimized are shown in Table 4.

Many design elements on the overview screens and the educational features were rated positively by older adults. For educational content with texts, the font sizes, readability, and length of the text were positively rated in almost all corresponding tasks (92.3%).

Moreover, more than half of the participants (25/49, 51%) were able to successfully solve more than 90% of the tasks. Exploratory analysis of group differences suggested that those who solved more than 90% of the tasks had significantly higher technology affinity (*P*=.02). The participants who were able to solve more than 90% of the tasks also rated the e-coach significantly better with the SUS score (*P*=.04).

**Table 4:** Content and structures optimized following iterative testing with participants.

Domain	Adaptations
Navigation	On the main overview screens for the nutrition and mobility modules, details on the content of modules were added. Checkboxes for the confirmation of exercise execution and the labeling of the elements were enlarged. For screens that guide different topics in a module, a question or more guidance about the content was added in addition to the title (eg, increasing activity: How can I become more active in everyday life?).
Comprehension	Keywords were supplemented with further information (eg, *nutrition* was changed to *nutrition topics*). The wording *macronutrients* was changed to *nutrients*. An instruction for the action was added below the screen heading (eg, “Please select one of the following topics to get more information.”). Information for food groups was added (*2/5* was changed to *2 of 5 servings*).
Design	The symbols for nutrients (the molecule symbol was changed to a magnifying glass), interesting information (the light bulb was changed to a book with light bulb on it), and the nutrition diary (the booklet was changed to a book) were replaced. Photographs for text elements were exchanged for symbols or drawings. Exercise photos were used instead of exercise drawings; a white background was added to the exercise photos. Any other elements besides diagrams were removed from the evaluation screens. Feedback on reaching the training goal using flowers instead of stars was added (flowers contain additional information about the number of exercises performed).

### Evaluation of the SUS

The evaluation of the SUS showed a continuous improvement in the usability of the e-coach (Table 5).

Because the normality assumptions of the ANOVA were violated, a 1-way Welch-ANOVA was performed to determine whether the SUS score was significantly different among the test phases. The improvement in the SUS score was not statistically significant among the 3 test phases (Welch *F*_2,46_=1.79; *P*=.19).

**Table 5:** System Usability Scale (SUS) score for each iterative test phase.

Phase	Values		
	n (%)	Mean (SD; range)	Median (IQR)
Iterative phase 1	21 (43)	69.3 (16.3; 42.5-97.5)	65.0 (57.5-83.8)
Iterative phase 2	16 (33)	70.3 (18.7; 20.0-95.0)	77.5 (60.0-84.4)
Iterative phase 3	12 (25)	78.1 (11.8; 50.0-92.5)	82.5 (70.6-86.9)

## Discussion

### Principal Findings

We showed that it is possible to conduct iterative test phases and improve the usability of a health app, even for older adults with health restrictions who are undergoing inpatient rehabilitation. After 3 iterative test phases, the average SUS increased from 69.3 (SD 16.3; median 65) to 78.1 (SD 11.8; median 82.5), indicating good usability of the e-coach and is comparable with the SUS results on eHealth application use in other studies with older adults [45-47]. More hints were required to answer comprehension questions (20%-30.5%) than to solve navigation tasks (14.1%-26.5%) during testing. With minor support from the study team, by hints, it was almost always possible for participants to solve the tasks or questions. Overall, only 5.5% of all tasks in all tests could not be successfully completed. These results show that even participants who had greater difficulties using the e-coach were able to solve most tasks with minor support.

Comparing the completion of tasks among the 3 iterations, it is noticeable that in the first iterative test phase, hints were needed, especially for comprehension questions (30.5%), and a few tasks were not fulfilled by all participants in this test (1.9%). In this iterative phase, hints were often needed for comprehension questions, such as what can be done on certain screens and also concerning the interpretation of what was meant by certain titles (eg, an overview of the recommended amount of physical activity). These difficulties were solved by adding more information to the descriptive texts and partly by changing the wording of individual terms.

In the second iterative test phase, the proportion of required hints in the navigation tasks (26.5%) increased noticeably compared with the first iterative test phase (14.1%), and 15.2% of all navigation tasks could not be solved by the participants in the second iterative test phase. In the first iterative test phase, many navigation tasks included easier functions, such as navigation to the next screen, and only a few tasks with more complex navigation steps. In contrast, in the second iterative test phase, more complex tasks were added, requiring, for example, 2 navigation steps, and the proportion of simpler navigation tasks decreased accordingly (Table 1). It seems possible that the increase in the complexity of the navigation tasks explains the increase in the number of hints required, as well as the increase in the proportion of navigation tasks that could not be solved. However, we did not perform any corresponding measurements that would allow conclusions to be drawn about the participants’ working memory capacity, as this was not part of our study.

In the third iterative test phase, hints were also required more often for navigation tasks that required several navigation steps. Although there were some tasks in the last iteration phase that required more than one navigation step, only 17% (2/12) of all participants needed help with all navigation tasks, and 4.3% of all navigation tasks in the third iterative test phase were not successfully completed. However, in the comprehension tasks, the need for hints was almost similar in the last iterative phase (20%) compared with the second iterative phase (21.6%), but more tasks could not be solved (2.5%) than in the second iterative phase. Compared with the first iterative test phase, almost all participants (10/12, 83%) in the last iterative test phase were able to derive the following content from the labeling of the modules. In addition, the question about what can be done on the overview screens was also answered correctly by all participants. Nevertheless, some participants found it difficult to interpret diagrams, meaning that they required hints, and one participant was unable to solve the task.

Other aspects should be considered as possible reasons for the different performance in the tasks, in addition to the increase in complexity. There were fewer tasks and fewer participants in the third iterative test phase because increasing COVID-19 case numbers forced us to reduce contact times with the participants. In addition, all the comprehension questions that could not be answered successfully in the last test were questions about protein and energy intake shown in a diagram. This topic could be difficult for people with little existing knowledge of the subject.

Furthermore, in our exploratory analysis of group differences between half of the participants who solved more than 90% of the tasks correctly and those who solved less than 90% of the tasks correctly, we found indications that those who solved more than 90% of the tasks correctly had a significantly higher affinity for technology (*P*=.02). A significant correlation between the use of health apps by older people and a higher affinity for technology has also been found in other studies [9,48]. Moreover, a stronger interest in technology appears to have a significant influence on the use of information and communication technologies even among people aged >80 years [49]. To estimate how well older adults can cope with a health app or whether more support might be needed, technology affinity could be surveyed beforehand and used as an indicator of the need for support. Different variables such as education level, sex, and computer literacy did not significantly influence the use of health apps in the study by Rasche et al [9]. Age differed significantly among groups. Individuals who reported using health apps were significantly younger than those who did not use health apps. In contrast, Schlomann et al [49] also found no significant influence of the variable age among older adults who already used mobile devices (smartphones, tablets, fitness trackers, or smartwatches). In our study, individuals who correctly solved more than 90% of the tasks were younger (mean 76.0, SD 4.3 years) than those who correctly solved less than 90% of the tasks (mean 79.7, SD 7.4 years) but this difference was not statistically significant (*P*=.09).

Technology commitment in our study population was at a median of 3.5 (SD 0.6) points in the group of participants who correctly solved more than 90% of the tasks and at a median of 3.0 (0.6) in the group of people who correctly solved less than 90% of the tasks. This value seems to correspond approximately to the technology readiness of people in this age group in Germany. Rasche et al [9] found comparable values among older people who reported no app use (mean 2.9, SD 0.6 points) or app use but no health app use (mean 3.4, SD 0.6 points).

To increase the usability of e-coaches in health care, as well as for less technically inclined older persons, detailed instructions on how to use the e-coach could be applied. Older people seem to have greater benefits from step-by-step instructions when learning to use new technologies. To address this problem in a real-world setting in terms of use of the e-coach by older adults, guidance (eg, in the form of a printed manual) should be additionally offered [50]. Furthermore, it was helpful for participants if the screens contained additional information, for example, what should be done on the screen or what content and information they would receive if they selected certain modules. The screens were not rated as overly cluttered despite additional information. This may be an important fact for other researchers developing apps for multi-morbid older adults in rehabilitation facilities, who have rarely been involved in app development to this extent. Although studies on eHealth interventions are already being conducted in the context of geriatric rehabilitation, apps have only been considered as a form of intervention in a few studies to date, and many applications relate to specific clinical conditions, particularly in the neurological field [14]. In older adults with limited mobility and after hip fractures, positive effects on mobility, functional outcomes, and hospitalization could be achieved through telerehabilitation interventions with an app [16] or via web sites [15,51]. However, these applications do not focus on educating participants but rather on teaching exercises. People who have not yet reached this phase in their behavioral change process may therefore benefit less from these tools. Furthermore, it is possible to use apps to conduct relevant assessments for older people with multiple morbidities with regard to fall risk [46] and mobility [47]. The usability of these assessment apps was investigated in older people and achieved SUS scores of 77.9 and 77.6, comparable with the score gained by our e-coach. In general, there are already different apps in the rehabilitation context for postoperative care after certain surgical interventions [52] or for improving self-management of hypertension or for medication planning. In these studies, however, it became clear that apps from the app store often lacked an evidence-based background, usability for older people is poor, and there is also a lack of data security [53,54]. In the field of nutrition apps, no studies involving participants aged >70 years have been found in a review from 2019 [18].

The data on the TTM phases of the participants demonstrate that geriatric patients in rehabilitation are at different phases of the behavioral change process. In the iterative tests with nutrition content as well as with physical activity content, the proportion of participants in the first 2 phases of TTM (precontemplation and contemplation) and the remaining phases (preparation, action, and maintenance) was quite balanced. As such, the readiness and implementation of behavior change in the areas of nutrition and physical activity appear to be heterogeneous among the target group, requiring the use of different strategies in the e-coach to support behavior changes. No indications of significant group differences between the half of the participants who were able to solve more than 90% of their tasks and those who were able to solve less than 90% of the tasks were found for the TTM phase distribution in the area of nutrition. For the TTM phase in the physical activity domain, no testing for group differences was conducted because of the small sample size resulting from missing data from the last iterative test. A correlation between certain phases of behavior change and the ability to successfully solve tasks could be explained only to a limited extent. In the case of tasks for operating the app, such as finding specific screens or using buttons, personal readiness to change one’s nutritional or physical activity behavior should have little influence. It could be possible that the interpretation of specific content with terms such as *protein* or *food* *group* is more difficult for people with less existing knowledge. However, even someone without existing technical knowledge may have already achieved the nutrition and physical activity goals we used. Therefore, it is not practical to draw conclusions from the behavior change phase alone about the ability to use or the general understanding of the app based on our results.

When developing the e-coach, design recommendations for the target group [29,30] were considered in advance. The button labels and the texts on the navigation screens were phrased without technical terms to make them usable even for novice technology users. This, as well as the chosen high contrasts of the different contents and texts, was maintained throughout the development process. In some cases, the minimum size of the arrow buttons was exceeded, but based on the feedback from our tests, this did not represent a barrier for the patients.

In a few tests, it was noticed that a user wanted to select the correct button, but the pressure on the button was not recognized by the device. Besides a possible malfunction of the touchscreen, there are 2 points that have been described in further studies [30,55] and are also found in our study; these should also be considered with regard to other apps for this target group. One possible cause of failure could be the contact between the test person’s finger and the touchscreen. In addition, the conductivity of the skin decreases with age, but we also observed that some participants typed using their fingernails rather than their fingertips. To improve usability, older participants could alternatively be offered to operate the device with a stylus [30]. As a second possible source of error, we observed the button being pressed for a very long time or the finger already being positioned outside the button on the touchscreen and then moved toward the button. When training users, the use of buttons should be explained in more detail and practiced. Furthermore, feedback (haptic, auditory, or visual) could improve usability [30]; however, this additional function was not used in this study.

### Limitations

It is likely that more people who already had a general interest in nutrition, physical activity, or technology participated in the study. Therefore, a selection bias cannot be ruled out even if at least the measured technology commitment also corresponds to the figures from another study with older people in Germany with varying degrees of experience in the use of technology [9]. In addition, study participation was not dependent on specific criteria related to mobility limitation or malnutrition. In a recent review with meta-analysis on nutritional status and physical functionality of geriatric patients in rehabilitation, it was demonstrated that a large proportion of persons in geriatric rehabilitation are affected by malnutrition and mobility restrictions [3]. The results of our tests also confirmed this finding for the participants of this study. Malnutrition was present in 27% (13/49) of the participants, and the risk of malnutrition was present in 51% (25/49) of the participants, as measured by the MNA-SF. The PASE score was 46.2, which is significantly lower than the average score of community-dwelling older adults reported by Washburn et al [56].

Data collection was conducted by study team members who were involved in the development of the e-coach. An uninvolved person would have had to conduct usability tests to increase the objectivity of these tests. This was not possible because of the financial resources of the project.

Moreover, the test situation itself may have biased the results. On the one hand, it is likely that older adults were more nervous and made more mistakes than if they had used the e-coach unobserved. On the other hand, the possibility of receiving hints and help from the examiner could also have led to a situation in which help was requested more quickly and, if necessary, tasks would have been solved after some time without the help of the study team member. In addition, it cannot be ruled out that unintentional nonverbal responses were given by the study team (eg, nodding) that were not recorded in the documentation.

In the context of this study, the time spent on the task was not determined. The time it takes a participant to complete a task can be an indicator of usability, as quick performance can also indicate ease of use. However, there is also evidence from a study by Sonderegger et al [57] that the time to complete a task is less related to perceived usability in older people than in younger people. We decided not to measure the time during tasks so as not to create additional pressure in the already unfamiliar test situation and because time-critical aspects presumably tend to play a minor role in the use by our target group.

This study only evaluated whether e-coach elements are generally usable for older adults. It provides an indication of the e-coach’s usability for first-time users but not for a longer period of use.

### Conclusions

This study involved older people undergoing inpatient rehabilitation in iterative optimization and usability testing for an e-coach according to the German International Organization for Standardization 9241-210:2019 *Ergonomics of human–system interaction—Part 210: Human-centered design of interactive systems* [24] and the user-centered design process [23]. It has been shown that this approach can be successfully applied to this vulnerable and low technologically skilled target group. The involvement of the target group was very important in developing a program that older people could rely on that is oriented to their needs, that is based on a psychological model for long-term behavior change, and that can also be used by them. When an app addresses important health issues (eg, malnutrition and inactivity), it seems particularly important to consider known barriers such as a lack of confidence in the app, low usability, or even users’ low existing technical experience and to offer evidence-based support [9].

As the target group is particularly vulnerable, and an individual’s willingness to continuously use the e-coach may impose an additional burden on them, it is essential to evaluate the acceptance, willingness, and adherence for long-term use of the system in a further study. Previous studies have shown that it can be very difficult to recruit patients for such long-term use of technical devices in this target group, and that good usability, as well as subjective benefits for patients, must necessarily be present [58].

## Appendix

Multimedia Appendix 1 Examples of tasks in iterative phase 2 physical activity.

Multimedia Appendix 2 Examples of tasks in iterative phase 3 nutrition.

Multimedia Appendix 3 Unsolved tasks and optimizations.

## Abbreviations

MNA-SF: Mini Nutritional Assessment-Short Form
PASE: Physical Activity Scale for the Elderly
SUS: System Usability Scale
TTM: transtheoretical model of behavior change

## References

[1] Benzinger P, Eidam A, Bauer JM (2021). [Clinical importance of the detection of frailty]. Z Gerontol Geriatr.

[2] Nascimento CM, Ingles M, Salvador-Pascual A, Cominetti MR, Gomez-Cabrera MC, Viña J (2019). Sarcopenia, frailty and their prevention by exercise. Free Radic Biol Med.

[3] Wojzischke J, van Wijngaarden J, van den Berg C, Cetinyurek-Yavuz A, Diekmann R, Luiking Y, Bauer J (2020). Nutritional status and functionality in geriatric rehabilitation patients: a systematic review and meta-analysis. Eur Geriatr Med.

[4] van Balen R, Gordon AL, Schols JM, Drewes YM, Achterberg WP (2019). What is geriatric rehabilitation and how should it be organized? A Delphi study aimed at reaching European consensus. Eur Geriatr Med.

[5] Grund S, van Wijngaarden JP, Gordon AL, Schols JM, Bauer JM (2020). EuGMS survey on structures of geriatric rehabilitation across Europe. Eur Geriatr Med.

[6] Hill A, Hoffmann T, McPhail S, Beer C, Hill KD, Brauer SG, Haines TP (2011). Factors associated with older patients' engagement in exercise after hospital discharge. Arch Phys Med Rehabil.

[7] Forkan R, Pumper B, Smyth N, Wirkkala H, Ciol MA, Shumway-Cook A (2006). Exercise adherence following physical therapy intervention in older adults with impaired balance. Phys Ther.

[8] Bitkom (2020). Welche der folgenden Gesundheits-Apps nutzen Sie bereits bzw. können Sie sich vorstellen, zukünftig zu nutzen?. Statista.

[9] Rasche P, Wille M, Bröhl C, Theis S, Schäfer K, Knobe M, Mertens A (2018). Prevalence of health app use among older adults in Germany: national survey. JMIR Mhealth Uhealth.

[10] Seifert A, Meidert U (2018). Quantified seniors. Technically assisted self-measurement among older adults. Präv Gesundheitsf.

[11] Mehra S, Visser B, Dadema T, van den Helder J, Engelbert RH, Weijs PJ, Kröse BJ (2018). Translating behavior change principles into a blended exercise intervention for older adults: design study. JMIR Res Protoc.

[12] Bickmore TW, Silliman RA, Nelson K, Cheng DM, Winter M, Henault L, Paasche-Orlow MK (2013). A randomized controlled trial of an automated exercise coach for older adults. J Am Geriatr Soc.

[13] Silveira P, van de Langenberg R, van Het Reve E, Daniel F, Casati F, de Bruin ED (2013). Tablet-based strength-balance training to motivate and improve adherence to exercise in independently living older people: a phase II preclinical exploratory trial. J Med Internet Res.

[14] Kraaijkamp JJ, van Dam van Isselt EF, Persoon A, Versluis A, Chavannes NH, Achterberg WP (2021). eHealth in geriatric rehabilitation: systematic review of effectiveness, feasibility, and usability. J Med Internet Res.

[15] Ortiz-Piña M, Molina-Garcia P, Femia P, Ashe MC, Martín-Martín L, Salazar-Graván S, Salas-Fariña Z, Prieto-Moreno R, Castellote-Caballero Y, Estevez-Lopez F, Ariza-Vega P (2021). Effects of tele-rehabilitation compared with home-based in-person rehabilitation for older adult's function after hip fracture. Int J Environ Res Public Health.

[16] Bean JF, Brown L, DeAngelis TR, Ellis T, Kumar VS, Latham NK, Lawler D, Ni M, Perloff J (2019). The rehabilitation enhancing aging through connected health prehabilitation trial. Arch Phys Med Rehabil.

[17] Best R, Souders DJ, Charness N, Mitzner TL, Rogers WA (2015). The role of health status in older adults' perceptions of the usefulness of eHealth technology. Hum Asp IT Aged Popul (2015).

[18] Villinger K, Wahl DR, Boeing H, Schupp HT, Renner B (2019). The effectiveness of app-based mobile interventions on nutrition behaviours and nutrition-related health outcomes: a systematic review and meta-analysis. Obes Rev.

[19] (2020). Ergonomics of human-system interaction - Part 210: Human-centred design for interactive systems (ISO 9241-210:2019); German version EN ISO 9241-210:2019. Beuth Publishing DIN.

[20] Richter M, Flückiger M (2013). Usability Engineering Kompakt: Benutzbare Produkte Gezielt Entwickeln.

[21] Happe L, Hein A, Diekmann R (2021). What do geriatric rehabilitation patients and experts consider relevant? Requirements for a digitalised e-coach for sustainable improvement of nutrition and physical activity in older adults - a qualitative focus group study. BMC Geriatr.

[22] Warschburger P, Warschburger P (2009). Beratungspsychologie.

[23] Romain A, Bortolon C, Gourlan M, Carayol M, Decker E, Lareyre O, Ninot G, Boiché J, Bernard P (2018). Matched or nonmatched interventions based on the transtheoretical model to promote physical activity. A meta-analysis of randomized controlled trials. J Sport Health Sci.

[24] DiClemente CC, Prochaska JO, Gibertini M (1985). Self-efficacy and the stages of self-change of smoking. Cogn Ther Res.

[25] Prochaska JO, Butterworth S, Redding CA, Burden V, Perrin N, Leo M, Flaherty-Robb M, Prochaska JM (2008). Initial efficacy of MI, TTM tailoring and HRI's with multiple behaviors for employee health promotion. Prev Med.

[26] Jiménez-Zazo F, Romero-Blanco C, Castro-Lemus N, Dorado-Suárez A, Aznar S (2020). Transtheoretical model for physical activity in older adults: systematic review. Int J Environ Res Public Health.

[27] Brooke J, Jordan PW, Weerdmeester B, Thomas A, McIelland IL (1996). SUS: A 'Quick and Dirty' usability scale. Usability Evaluation in Industry.

[28] Faulkner L (2003). Beyond the five-user assumption: benefits of increased sample sizes in usability testing. Behavior Research Methods, Instruments, & Computers.

[29] Eichhorn C, Plecher D, Lurz M, Leipold N, Böhm M, Krcmar H, Ott A, Volkert D, Hiyama A, Klinker G (2020). Combining motivating strategies with design concepts for mobile apps to increase usability for the elderly and Alzheimer patients. HCII 2020: Human Aspects of IT for the Aged Population. Healthy and Active Aging.

[30] Nurgalieva L, Laconich JJ, Baez M, Casati F, Marchese M (2019). A systematic literature review of research-derived touchscreen design guidelines for older adults. IEEE Access.

[31] Volkert D, Beck AM, Cederholm T, Cruz-Jentoft A, Goisser S, Hooper L, Kiesswetter E, Maggio M, Raynaud-Simon A, Sieber CC, Sobotka L, van Asselt D, Wirth R, Bischoff SC (2019). ESPEN guideline on clinical nutrition and hydration in geriatrics. Clin Nutr.

[32] Deutsche Gesellschaft für Ernährung e. V. (2014). DGE Praxiswissen: Essen und Trinken im Alter. Fit Im Alter: Gesund Essen, Besser Leben.

[33] Deutsche Gesellschaft für Ernährung e.V. (2012). DGE Praxiswissen. Mangelernährung im Alter. Fit Im Alter: Gesund Essen, Besser Leben.

[34] Bundeszentrale für gesundheitliche Aufklärung (2015). Aktiv im Alltag, aktiv im Leben: Fit und eigenständig bleiben: Anregungen für Menschen mit Bewegungseinschränkungen. Älter Werden in Balance.

[35] Liu-Ambrose T, Donaldson MG, Ahamed Y, Graf P, Cook WL, Close J, Lord SR, Khan KM (2008). Otago home-based strength and balance retraining improves executive functioning in older fallers: a randomized controlled trial. J Am Geriatr Soc.

[36] Clemson L, Singh MA, Bundy A, Cumming RG, Manollaras K, O'Loughlin P, Black D (2012). Integration of balance and strength training into daily life activity to reduce rate of falls in older people (the LiFE study): randomised parallel trial. Br Med J.

[37] Clegg A, Barber S, Young J, Iliffe S, Forster A (2014). The Home-based Older People's Exercise (HOPE) trial: a pilot randomised controlled trial of a home-based exercise intervention for older people with frailty. Age Ageing.

[38] Kaiser MJ, Bauer JM, Ramsch C, Uter W, Guigoz Y, Cederholm T, Thomas DR, Anthony P, Charlton KE, Maggio M, Tsai AC, Grathwohl D, Vellas B, Sieber CC, MNA-International Group (2009). Validation of the Mini Nutritional Assessment short-form (MNA-SF): a practical tool for identification of nutritional status. J Nutr Health Aging.

[39] Deutsche Gesellschaft für Ernährung e. V. (2020). Dge-qualitätsstandard Für Die Verpflegung Mit Essen Auf Rädern Und in Senioreneinrichtungen.

[40] Washburn R, Smith K, Jette A, Janney C (1993). The physical activity scale for the elderly (PASE): development and evaluation. J Clin Epidemiol.

[41] Pfeifer K, Rütten A (2017). [National recommendations for physical activity and physical activity promotion]. Gesundheitswesen.

[42] Neyer FJ, Felber J, Gebhardt C (2012). Entwicklung und Validierung einer Kurzskala zur Erfassung von Technikbereitschaft. Diagnostica.

[43] Silva P, Nunes F (2010). 3 x 7 usability testing guidelines for older adults. Proceedings of the 3rd Mexican Workshop on Human Computer Interaction (MexIHC'2010).

[44] Ericsson K, Simon HA (1984). Protocol Analysis : Verbal Reports as Data.

[45] Bangor A, Kortum P, Miller J (2009). Determining what individual SUS scores mean: adding an adjective rating scale. J Usab Stud Arch.

[46] Hsieh KL, Fanning JT, Rogers WA, Wood TA, Sosnoff JJ (2018). A fall risk mHealth app for older adults: development and usability study. JMIR Aging.

[47] Bergquist R, Vereijken B, Mellone S, Corzani M, Helbostad JL, Taraldsen K (2020). App-based self-administrable clinical tests of physical function: development and usability study. JMIR Mhealth Uhealth.

[48] Seifert A, Vandelanotte C (2021). The use of wearables and health apps and the willingness to share self-collected data among older adults. Aging Health Res.

[49] Schlomann A, Seifert A, Zank S, Rietz C (2020). Assistive technology and mobile ICT usage among oldest-old cohorts: comparison of the oldest-old in private homes and in long-term care facilities. Res Aging.

[50] Hickman J, Rogers W, Fisk A (2007). Training older adults to use new technology. J Gerontol B Psychol Sci Soc Sci.

[51] Báez M, Ibarra F, Far I, Ferron M, Casati F (2016). Online group-exercises for older adults of different physical abilities. Proceedings of the International Conference on Collaboration Technologies and Systems (CTS).

[52] Patel B, Thind A (2020). Usability of mobile health apps for postoperative care: systematic review. JMIR Perioper Med.

[53] Alessa T, Hawley MS, Hock ES, de Witte L (2019). Smartphone apps to support self-management of hypertension: review and content analysis. JMIR Mhealth Uhealth.

[54] Stuck RE, Chong AW, Mitzner TL, Rogers WA (2017). Medication management apps: usable by older adults?. Proc Hum Factors Ergon Soc Annu Meet.

[55] Kobayashi M, Hiyama A, Miura T, Asakawa C, Hirose M, Ifukube T, Campos P, Graham N, Jorge J, Nunes N, Palanque P, Winkler M (2011). Elderly user evaluation of mobile touchscreen interactions. Human-Computer Interaction – INTERACT 2011.

[56] Washburn RA, McAuley E, Katula J, Mihalko SL, Boileau RA (1999). The physical activity scale for the elderly (PASE): evidence for validity. J Clin Epidemiol.

[57] Sonderegger A, Schmutz S, Sauer J (2016). The influence of age in usability testing. Appl Ergon.

[58] Nymberg VM, Bolmsjö BB, Wolff M, Calling S, Gerward S, Sandberg M (2019). 'Having to learn this so late in our lives…' Swedish elderly patients' beliefs, experiences, attitudes and expectations of e-health in primary health care. Scand J Prim Health Care.

