# Supplementary figures and images for: Iterative Development and Applicability of a Tablet-Based e-Coach for Older Adults in Rehabilitation Units to Improve Nutrition and Physical Activity: Usability Study
Source: JMIR Hum Factors. 2022 Mar 16;9(1):e31823. doi: 10.2196/31823 (PMC8968623; doi:10.2196/31823)

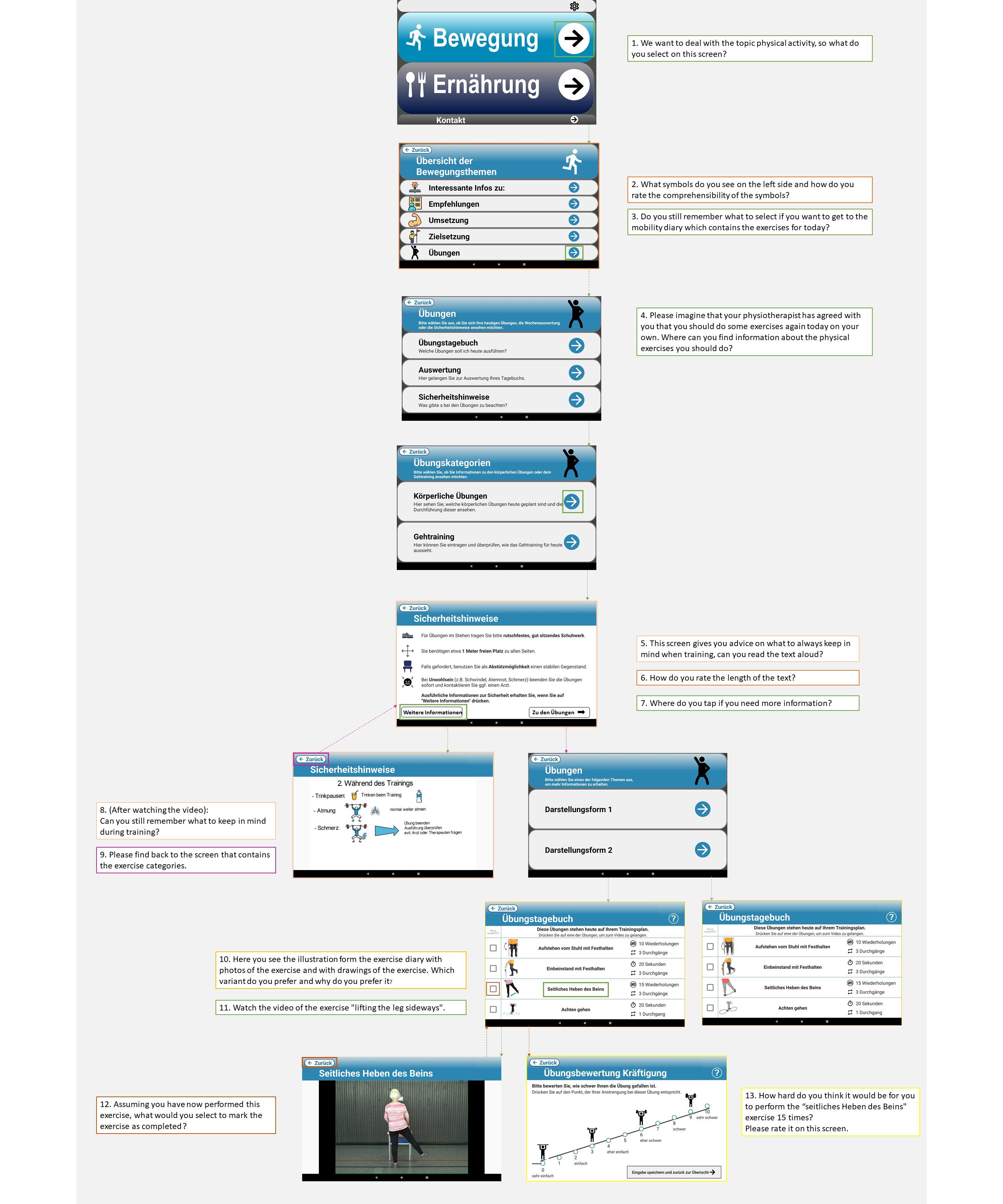

Supplement: Multimedia Appendix 1 [file humanfactors_v9i1e31823_app1.png]

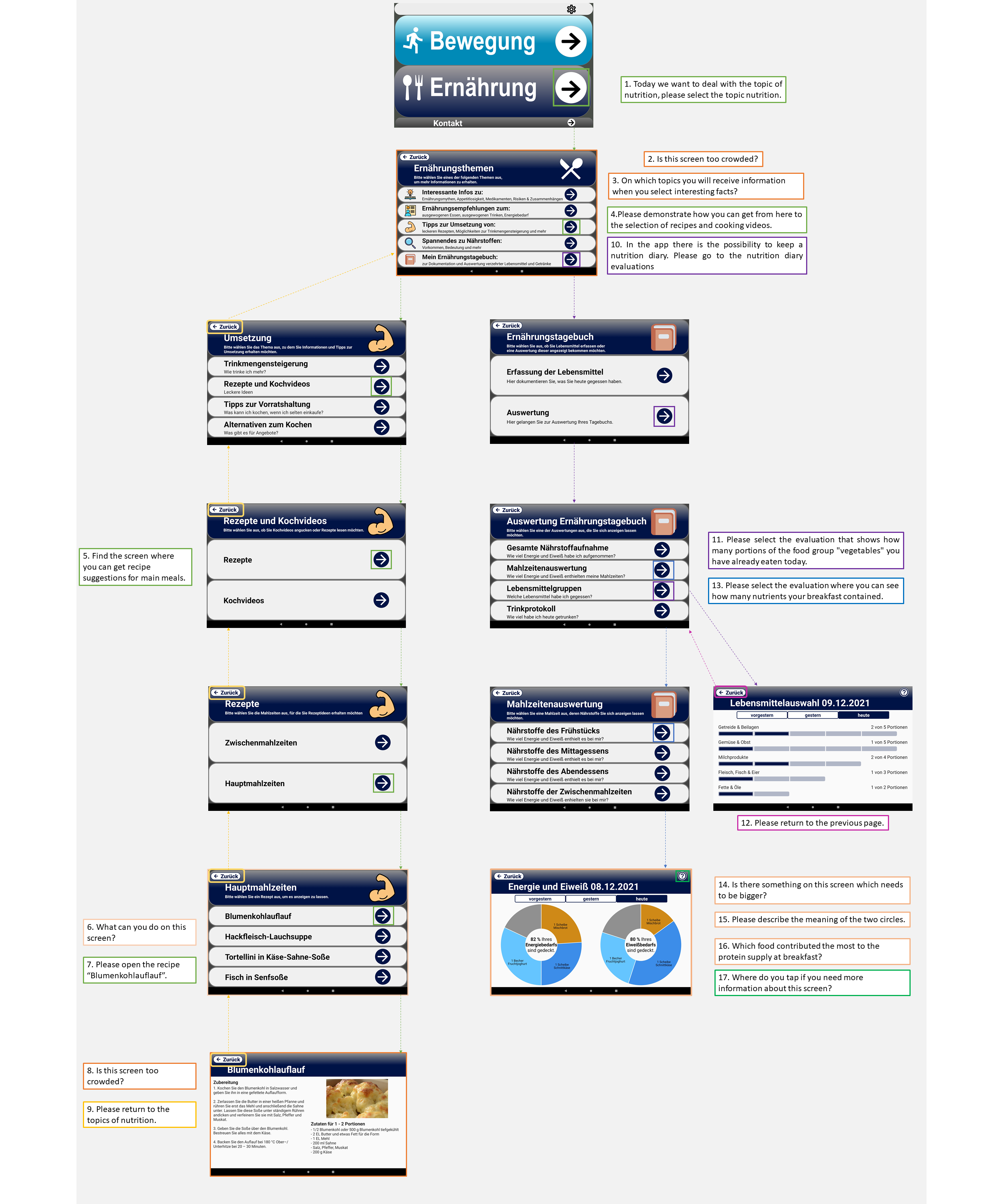

Supplement: Multimedia Appendix 2 [file humanfactors_v9i1e31823_app2.png]
