# Supplementary material for: Iterative Development and Applicability of a Tablet-Based e-Coach for Older Adults in Rehabilitation Units to Improve Nutrition and Physical Activity: Usability Study
Source: JMIR Hum Factors. 2022 Mar 16;9(1):e31823. doi: 10.2196/31823 (PMC8968623; doi:10.2196/31823)
Supplement: Multimedia Appendix 3 [file humanfactors_v9i1e31823_app3.pdf]

Iterative Phase	Task type	Screen<br>Question	Optimisation
1	Use of the nutrition diary	Nutrition diary:<br>Enter the following foods at breakfast:<br>- 1 glass of water<br>- 1 slice of mixed bread<br>- 1 slice of sliced cheese<br>- 1 cup fruit yoghurt 1,5%<br>Enter the following foods at lunch<br>- 1 handful broccoli<br>- 1 handful of boiled or jacket potatoes<br>- 1 palm of low fat fish	None<br>(detailed description of the handling in the manual necessary)
2	Navigation further screen (≥ 2)	Overview screen nutrition:<br>Do you still remember what to select in order to find the information about the connection between medications and appetite?	Details on the content of modules were added (see Figure 2)
		Overview nutrition themes:<br>In the app there is the option to keep a nutrition diary. I have already done that today. Can you show me where I can look at my evaluation of what I ate?	
	Navigation next screen (1 screen)	Overview of interesting facts-medication:<br>Please imagine that you were given a new medication and after a few weeks you noticed that your appetite was dropping. Where do you tap to see whether the new medication might is responsible?	At screens that guide to different topics in a module, a question or more guidance about the content was added or the wording was: e.g. Medication - Influence of medication on appetite.
		Overview of interesting facts-medication:<br>Now please imagine that you are prescribed an antibiotic and you are unsure whether you can still eat dairy products. Where do you look now?	
		Overview of evaluations:<br>Can you show me where to find the evaluation which says how many portions of vegetables I have eaten?	
		Safety instruction screen:<br>Where do you tap if you need more information?	Text colour of the button was changed for better visibility.

<table>
<tr><td rowspan="5"></td><td rowspan="2">Use the help button</td><td>Information on influence of medication on appetite:
Where do you tap if you do not know about what you are being informed here?</td><td rowspan="2">Design of the help button was changed (Question mark is now blue and has a bold white circle around it for easier identification as a button)
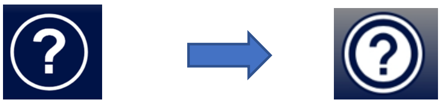</td></tr>
<tr><td>Finding the help screen:
Where do you tap if you do not understand what this screen is supposed to tell</td></tr>
<tr><td rowspan="2">Use of back button</td><td>Information on influence of medication on appetite:
Please navigate back to the start, to the selection option between nutrition and mobility</td><td rowspan="2">None.
Detailed description of the handling in the manual necessary.</td></tr>
<tr><td>Overview nutrition themes:
Please navigate to the previous page.</td></tr>
<tr><td>Use of exercise diary</td><td>Exercise diary- confirmation of exercise:
Assuming you have now performed this exercise, what would you select to mark the exercise as completed?</td><td>Colour of checkboxes for confirmation of exercise execution was changed and slightly enlarged. Labelling of the confirmation instruction was enlarged.
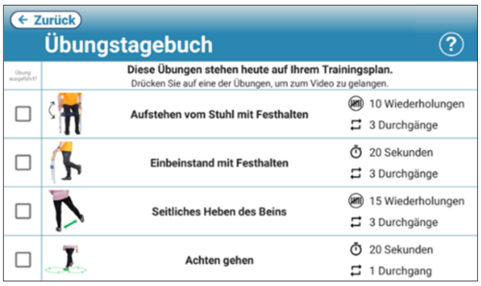

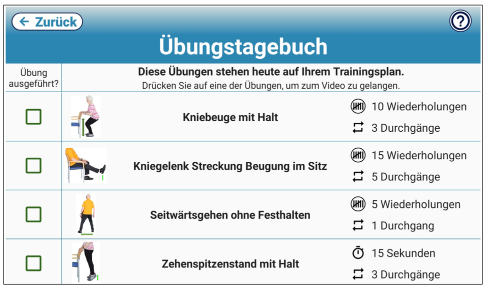
</td></tr>
<tr><td rowspan="2">3</td><td rowspan="2">Navigation further screen (≥ 2)</td><td>Overview nutrition topics:
Please show me where you can find recipe suggestions for main meals.</td><td rowspan="2">None.
Detailed description of the handling in the manual necessary.</td></tr>
<tr><td>Overview of nutrition diary evaluation:
Please select the evaluation where you can see how many nutrients your breakfast contained.</td></tr>
</table>

	Navigation next screen (1 screen)	Overview of nutrition diary evaluation: Please select the evaluation that shows how many portions of the food group "vegetables" you have already eaten today.	None.<br>Detailed description of the handling in the manual necessary.<br>Detailed description of the handling in the manual necessary.
	Use of back button	Evaluation of food groups: Please return to the previous page.	In the screen header, the title has been centered, visually creating more space between the back button and the title.
	Interpretation of content	Evaluation of protein and calorie at breakfast: Please describe the meaning of the two charts	Title of screen was changed, now providing the date and kind of meal that is displayed (breakfast, lunch, dinner, snack). Bar charts have been implemented in the meal evaluation to ensure the clarity of the charts when many food items are entered.<br>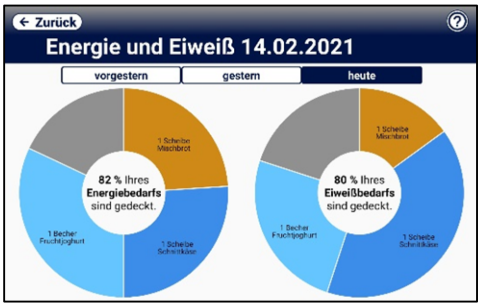<br>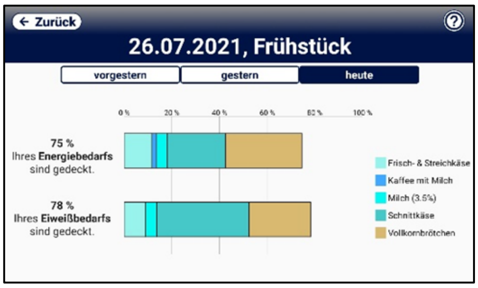
	Use the help button	Evaluation of protein and calorie at breakfast: Where do you tap if you need more information about this screen?	In the screen header, the title has been centered, visually creating less space between the back button and the title.
